# Taking up Cancer Immunotherapy Challenges: Bispecific Antibodies, the Path Forward?

**DOI:** 10.3390/antib5010001

**Published:** 2015-12-26

**Authors:** Joanie Del Bano, Patrick Chames, Daniel Baty, Brigitte Kerfelec

**Affiliations:** 1 Inserm, U1068, CRCM, Marseille F-13009, France; joanie.del-bano@inserm.fr (J.D.B.); patrick.chames@inserm.fr (P.C.); daniel.baty@inserm.fr (D.B.); 2 Aix-Marseille University, Marseille F-13284, France; 3 CNRS, UMR7258, CRCM, Marseille F-13009, France; 4 Institut Paoli-Calmettes, Marseille F-13009, France

**Keywords:** bispecific antibody, cancer immunotherapy, NK cells, T-cells, immune effector cells, immuno-checkpoint

## Abstract

As evidenced by the recent approvals of Removab (EU, Trion Pharma) in 2009 and of Blincyto (US, Amgen) in 2014, the high potential of bispecific antibodies in the field of immuno-oncology is eliciting a renewed interest from pharmaceutical companies. Supported by rapid advances in antibody engineering and the development of several technological platforms such as Triomab or bispecific T cell engagers (BiTEs), the “bispecifics” market has increased significantly over the past decade and may occupy a pivotal space in the future. Over 30 bispecific molecules are currently in different stages of clinical trials and more than 70 in preclinical phase. This review focuses on the clinical potential of bispecific antibodies as immune effector cell engagers in the onco-immunotherapy field. We summarize current strategies targeting various immune cells and their clinical interests. Furthermore, perspectives of bispecific antibodies in future clinical developments are addressed.

## 1. Introduction

Understanding the intimate relations between the immune system and the carcinogenesis process is an old research line in immunology. In the late nineteenth century, Coley had indeed demonstrated that infection triggered by bacterial toxins-based treatment promoted tumor regression by stimulation of the immune system [1,2]. Still, it remains a challenging field of research as new interrelations are continuously discovered and the more we know, the more complex it looks.

Proposed in the mid-1950s by Burnet and Thomas [3,4], the cancer immunosurveillance hypothesis stated the protective role of the innate immune system in cancer. It underwent skepticism and several reconsiderations [5,6] for years before being finally validated in the 1990s. However, this concept did not fully summarize the paradoxical role of the immune system in oncogenesis [7], failing to explain how tumors achieved tolerance to the immune system. In the 2000s, the dual role of the immune system in cancer became evident: it plays a protective role by eliminating nascent malignant cells but also promotes malignant cells escape from immune response and elimination by shaping the immunogenicity of tumor cells [8,9,10,11].

These findings paved the way for a new concept of cancer immunoediting characterized by three dynamic phases: elimination, equilibrium and escape [8,10,12,13,14,15,16,17]. The elimination phase represents the original concept of cancer immunosurveillance whereby innate and adaptive immunities collaborate for protecting immunocompetent organisms from the development of tumor [18,19]. The equilibrium phase is triggered by the survival of some tumor cells after incomplete tumor destruction during elimination phase [20]. During this phase, the immune system maintains the tumor cells in a functionally dormant state and shapes the immunogenicity of the malignant cells through selective pressure. The final phase describes the tumor escape from the immunological control through several mechanisms and the outgrowth of tumor [8,9,10,12,21,22,23]. In this latter phase, the immune system is not the only one to blame as growing evidences demonstrate that tumors are able to shape their microenvironment for promoting their growth. Indeed, the cellular components of the tumor microenvironment, mainly non malignant cells such as fibroblasts and tumor-infiltrated immune cells, could be subjected to a functional switch towards tumor-promoting phenotypes driven by cellular cross-talks (paracrine communications or/and direct interaction with tumor cells) [24]. Accumulation of immunosuppressive cells and related cytokines induce the anergy of infiltrated cytotoxic immune cells, in particular effector T-cells [25]. Several studies on immune tumor infiltration have demonstrated a correlation between the patient’s overall outcome and the presence, the localization, the nature (T-lymphocytes [26,27], NK cells [28] or macrophages [29], dendritic cells (DC), B cells, *etc.*), and the functional status of immune infiltrated cells [30].

This immune contexture has been shown to be heterogeneous both between patients and between tumor types, thus influencing the anti-tumor immune responses [31]. Viewed in this light, immune contexture is now used as a therapeutic and prognostic marker in a new test called Immunoscore^®^ for a better stratification of patients, in addition to the Tumor-Node-Metastasis classification [32,33,34]. Thus, the ever expanding insight on the mechanisms underlying the dynamic interactions between the immune system and the tumor has changed the scientific perception of cancer. Until recently, cancer was seen as an anomaly of cell proliferation; it is now further defined as a dysfunction of the immune system promoted by tumor cells pressing down on the brakes (inhibitory receptors) that naturally exist in immune effector cells in order to prevent a runaway of the immune system [35]. The Graal of immunotherapy is thus to (re)stimulate the body’s own immune system for counteracting the camouflage of tumor cells and the tumor-induced immunosuppressive environment. For that purpose, several immune-based therapeutic strategies exist such as: (i) vaccine approaches to prime the immune system for eliciting a sustained immune responses. These approaches have achieved variable degrees of success and up to now, only one dendritic cell-based therapeutic vaccine, Provenge^®^ (sipuleucel-T) for prostate cancer treatment [36] has reached the market (FDA approval 2010); (ii) adoptive transfer of *ex vivo*-enriched and expanded tumor-specific T lymphocytes approaches [37,38]. CD19-directed CAR is one of the most promising chimeric antigen receptor (CAR-T) T-cell therapies conceived for treatment of Acute Lymphoblastic leukemia (ALL) [39]; (iii) monoclonal antibody-based therapies targeting either tumor antigens (rituximab [40], trastuzumab [41]) or immunosuppressive mechanisms on T-cells such as ipilimumab which blocks the immuno-checkpoint CTLA-4 (cytotoxic T-lymphocyte-associated antigen-4) [42].

Among all those immunotherapeutic strategies, monoclonal antibody-based therapeutics are currently the fastest growing segment of the drug and biological market. Since the approval of the first chimeric antibody, rituximab [43], in 1997, about 20 monoclonal antibodies (mAb) for cancer have been approved or in review in the USA and Europe [44], representing more than 50% of worldwide market of therapeutic mAbs. Tremendous efforts have been made to improve mAbs efficiency and especially their antibody-dependent cell-mediated cytotoxicity (ADCC) through (glyco)-engineering of crystallizable fragment (Fc) [45,46]. This led to the approval of second generation antibodies such as obinutuzumab [47], a low fucosylated anti-CD20 mAb that clearly outperforms rituximab, the first generation molecule.

Surfing on the edges of the clinical successes of mAbs and on the development of antibody engineering, new players such as bispecific antibodies are gaining ground among antibody-based therapeutics [48]. Bispecific antibodies (bsAbs) are antibody-derived constructs that combine the benefits of the binding specificities of two monoclonal antibodies into a single molecule [46,49]. Thus, bsAbs offer the opportunity to co-target two different epitopes on the same or different antigens. This concept emerged years ago, soon after scientists could reliably produce mAbs. However it remained dormant for years because of manufacturing problems and, most of all, low clinical benefit [46]. During the last decade, the bsAb field has made tremendous progress as evidenced by the recent clinical approvals of two bsAbs. Advances in recombinant DNA technology and bioengineering have led to the development of several technological platforms involved in the design of an impressive variety of bsAb formats [49,50,51].

The design of bsAb in the oncology field is focused on two main therapeutic approaches: (i) the first one aims at targeting simultaneously two receptors or two ligands for co-inhibiting redundant signaling pathways [52]; (ii) the second approach, which constitutes the main focus of this review, aims at harnessing and reawakening immune effector cells in the tumor microenvironment [53].

In this review, we give an update on the main preclinical and clinical results obtained with bispecific antibodies targeting T-cells and discuss current strategies targeting other types of immune cells such as, NK and macrophages, and their clinical potentials in order to take up the immunotherapy challenge.

## 2. Bispecific Antibody Technological Platforms

The idea of using bispecific antibodies to efficiently retarget effector immune cells toward tumor cells emerged in the 1980s [54,55,56]. First generation bsAbs were classically engineered either by chemical cross-linking or by somatic hybridization of two antibody-secreting hybridoma cells (quadromas technology) [57]. However, technological hurdles such as low yield, heterogeneity of batch production and immunogenicity caused by human anti-mouse/rat antibody (HAMA/HARA) strongly hindered the development of this first generation of bispecific antibodies until the late 1990s. A turning point was reached with bioengineering progress which has allowed the design of recombinant engineered antibody scaffolds that possess the full binding activity of the entire immunoglobulin G (IgG) molecule, and profoundly revolutionized the bsAb technology. Using these antibody fragments as building blocks to create multispecific/multifunctional molecules has led to a plethora of next-generation bispecific formats (>50) with their own benefits and drawbacks according to the desired application (immunotherapy, imaging, *etc.*). A detailed description of all the formats is beyond the scope of this review but several well-documented reviews have been recently published [50,51,58]. Bispecific scaffolds are generally classified in two major groups with different pharmacokinetic properties, based on the absence or presence of an Fc fragment, IgG-like molecules and small recombinant bispecific formats, most of them deriving from single chain variable fragment (scFv). Through their compact size, antibody fragments usually penetrate tumors more efficiently than IgG-like molecules but this benefit is mitigated by a short serum half-life (few hours) limiting their overall tumor uptake and residence time [59]. By contrast, the presence of an Fc fragment, which binds to the neonatal Fc receptors, provides a long serum half-life (>10 days) to the IgG-like formats, favoring tumor uptake and retention, but limits tumor penetration.

All these technological improvements prompted several biotech companies such as Trion Pharma, Amgen, Affimed, MacroGenics, Roche and many others to dedicate part of their research efforts in the development of technological platforms for designing bispecific antibodies.

**Figure 1:**
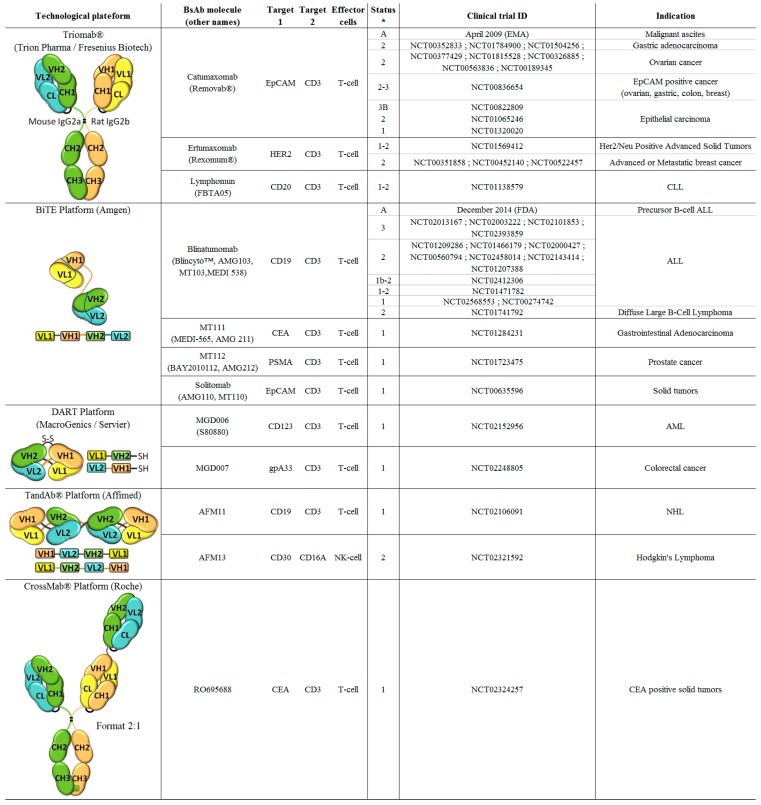
Bispecific antibodies targeting immune cells, approved or in clinical trials. A: Approved; 1, 2, 3: Phases of clinical trials.

In this part, we are presenting a non-extensive glimpse on bsAb technological platforms that develop bsAbs engaging immune cells for cancer therapy, focusing our attention on bsAb formats that reached the clinical arena (Figure 1).

### 2.1. Trifunctional Hybrid Antibodies Platform—Triomab^®^ Format (Trion Pharma (Fresenius Biotech, Planegg, Germany))

The Triomab^®^ format (150 kDa), introduced in 1995 by Lindhofer and collaborators, is a chimeric construction made of half of two full-length antibodies of different isotypes, mouse IgG2a and rat IgG2b [60]. This technology, which relies on a species-preferential heavy/light chain pairing association, has considerably improved the classical quadroma approach for producing bsAbs [60]. All candidates of the Triomab^®^ family are designed in the same way with an anti-CD3 rat IgG2b moiety and a mouse IgG2a moiety targeting various tumor-associated antigens (TAA). These chimeric IgG-like bsAbs are actually bispecific and trifunctional as they target tumor cells via TAA binding, T-cells via CD3 targeting and FcγR bearing immune cells (NK cells, macrophages or dendritic cells) via FcγR binding. Indeed, the hybrid mouse/rat Fc portion is able to bind and activate the human Fc receptors (FcγRI, FcγRIIA and FcγRIIIA) but not the inhibitory receptor FcγRIIB [61,62,63].

The beacon of this platform is Removab^®^ (Catumaxomab), an anti-CD3 × EpCAM (Figure 1).

### 2.2. Bispecific T-Cell Engager (BiTE) Platform (Amgen, Thousand Oaks, CA, US)

In the nineties, the Micromet Inc. company (now part of Amgen), taking advantage of the pioneering works of Mack and collaborators [54,64], developed the so-called BiTEs for Bispecific T-cell Engagers (BiTEs^®^). BiTEs (~50 kDa) are made by genetically fusing an anti-CD3ε scFv to an anti-TAA scFv via a flexible peptide linker (GGGGS). Compared to IgG-like format, BiTEs are endowed with a short serum half-life, due to their small size and the lack of Fc region, a characteristic that could be a potential advantage (tumor penetration, for instance) or a disadvantage (continuous infusion required).

Several BiTEs directed against a large variety of TAA such as EpCAM, HER2, EGFR, CEA, EphA2, CD33, and MCSP have been created by the BiTE platform [65]. The beacon of this platform is Blinatumomab, an anti-CD19 × CD3 (Figure 1).

### 2.3. Dual-Affinity Re-Targeting (DART) Platform MacroGenics (Rockville, MD, US)

The DART technology is based on the diabody format originally developed by Holliger *et al.* [66] and further improved for stability and optimal pairing of the V_H_ and V_L_ chains [67]. In contrast to BiTEs, DARTs (~50 kDa) are made of two polypeptide chains derived from the variable domains of two different antibodies (1 and 2) and covalently linked by a disulfide bridge. The first chain contains V_H_1 and V_L_2 and the second one, V_H_2 and V_L_1 (Figure 1). In each chain, the binding domains are connected by a short linker which, by impeding intra-chain pairing, promotes heterodimerization of the two chains.

### 2.4. TandAb^®^ Platform (Affimed, Heidelberg, Germany)

Like DARTs, TandAbs are based on the diabody concept but are designed as a single polypeptide chain VH1-VL2-VH2-VL1 comprising short linkers to prevent intra-chain pairing. Head-to-tail dimerization of this single chain leads to the formation of a tetravalent homodimer [68]. In contrast to the above-mentioned bsAb formats, TandAbs are bivalent for each specificity. With a molecular weight of 100–115 kDa, TandAbs developed by Affimed, have an increased plasma half-life compared to others small bispecific formats [68] while retaining a tumor penetration ability (Figure 1).

### 2.5. CrossMAb (Roche, Basel, Switzerland)

As Triomab, CrossMAb (150 kDa) are chimeric antibodies constituted by the halves of two full-length antibodies. For correct chain pairing, Roche has combined two technologies: (i) the well-known “knob-into-hole” which favors a correct pairing between the two heavy chains [69]; and (ii) an exchange between the heavy and light chains of one of the two Fabs to introduce an asymmetry which avoids light-chain mispairing (Figure 1) [70]. CrossMAbs can combine two or more antigen-binding domains for targeting two or more targets or for introducing bivalency towards one target such as the 2:1 format (Figure 1) [70,71].

## 3. T-Cells in bsAb-Mediated Immunotherapy

T-cells are the most abundant (~75% of blood lymphocytes) and potent immune killer cells. The role of effector T-cells in the anti-tumor immune response is strongly supported by *in vitro* studies and the observation that a high infiltration of CD8^+^ T cells in several types of tumors correlates with a favorable clinical prognostic [30].

One major limit of conventional monoclonal antibodies is their inability to target T-cells, as these cells do not express any Fcγ receptors. By contrast, the modulatory structure of bispecific antibodies offers the indisputable advantage of potentially targeting any type of immune effector cells, independently of the presence of Fcγ receptors. Thus, for obvious reasons, major research efforts have focused on the development of bispecific antibodies targeting effector T-cells. Using the T-cell receptor-CD3 complex (TCR-CD3) expressed at their surface, they constantly patrol the organism, seaking for “foreign” antigens issued from infected or transformed cells and presented by the MHC molecules of professional antigen presenting cells (APC) such as dendritic cells. The TCR is made of two polypeptide chains connected by a disulfide bridge, αβ for about 95% of blood T-cells, and γδ for about 5% of the T-cell population. The invariant CD3 molecules, made of 4 non-polymorphic polypeptides (ζ, δ, ε, γ), are non-covalently associated to the TCR and are involved in the intracellular signaling via their immunoreceptor tyrosine-based activation motifs (ITAMs). The fate of T-cells (maturation, activation, differentiation) is determined by the integration of signals derived from a close APC/T-cells contact. These signals are dependent on the type and activation state of APC, the frequency and duration of contacts and the identity and segregation of co-signaling molecules recruited in the immunological synapse [72,73]. Full activation of T-cells requires a temporary but long-lasting immune synapse for sustained signaling rather than short lived synapse that may induce tolerance.

The activation of effector naive T-cells requires at least three complementary signals: (i) TCR-CD3/Ag-MHC interaction with the assistance of co-receptors (CD4 or CD8); (ii) binding of co-stimulatory molecules such as CD80 or CD86 to CD28, CD40/CD40L; and (iii) accessory molecules such as cytokines. Actually, this rather schematic activation model has evolved with the discovery of new molecular interactions between T-cells and APC that modulate TCR signaling and the increasing identification of co-stimulatory receptors and co-inhibitory receptors [74].

### 3.1. CD3-TCR Targeting

**Figure 2:**
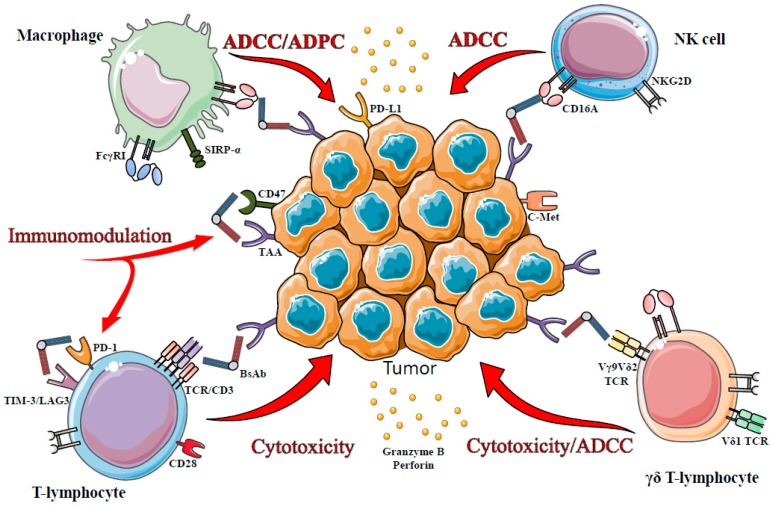
Immune effector cells targeted by bispecific antibodies. The mode of action of bispecific antibodies is related to the type of recruited immune cells: antibody-dependent cell-mediated cytotoxicity (ADCC) and antibody-dependent cellular phagocytosis (ADCP) are triggered by CD16A engagement on macrophages, NK cells or γδ T-cells; T-cell cytotoxicity is mediated by TCR/CD3 or Vγ9Vδ2 engagement or by blockade of immuno-checkpoints.

Targeting CD3 is particularly attractive (Figure 2) as it results in a polyclonal cytotoxic response bypassing the classical antigen-specific T-cell response [56] but raises some questionings: (i) all CD3^+^ T-cells will be indiscriminately stimulated including various immunoregulatory and immunosuppressive T-cells, which are described as playing an active role in immune evasion [30]; and (ii) CD3-mediated T cells activation in the absence of co-stimulatory signals has been shown to induce an unresponsiveness of T-cells concomitant to a loss of CD3 expression [75,76]. This discovery was at the origin of the clinical use of Muromomab, the first anti-CD3 monoclonal antibody approved by the FDA (1985, discontinued since 2010), to induce immunosuppression and reduce rejection in renal transplantation [77]. To avoid CD3-activated T cells anergy, anti-CD3 bispecific antibodies were first concomitantly administered with anti-CD28 antibodies but the combination yielded mitigated results [78]. However, the first clinical results of small bispecific formats such as BiTEs have alleviated these tricky concerns, even if the “how and why” questions are still not fully understood.

It is now well accepted that bsAbs-mediated killing of tumor cells by T-cells induces the formation of an artificial lytic synapse between the two cells which mimic the naturally occurring lytic synapses [79].

#### 3.1.1. From Bench to Bed Side: Two Bispecific Antibodies Approved for Human Use

##### Catumaxomab/Removab^®^

The pioneer bispecific antibody reagent in the market is catumaxomab, an anti-Epithelial Cell-Adhesion Molecule (EpCAM) × CD3 Triomab developed by Trion Pharma GmbH in collaboration with Fresenius Biotech (Figure 1). This bsAb is used for the intraperitoneal treatment of malignant ascites developed by patients with EpCAM^+^ carcinomas [80]. Malignant ascites are late-stage events that are associated with a poor prognosis and limited treatment options. EpCAM is a trans-membrane protein over-expressed all over the cell surface in a vast number of epithelial cancers that generate malignant effusions (70%–100%) [81,82,83]. Although expressed by the epithelium of healthy individuals, it is masked by tight junctions because of its baso-lateral localization and thus poorly accessible, an interesting advantage for an immunotherapy target. Its role in cancer is up for debate since it has been described both as an oncogene or as a tumor suppressive protein [84].

Preclinical studies have shown that catumaxomab can target epithelial EpCAM^+^ cells and human T-lymphocytes, and that the presence of the Fc fragment adds two major mechanisms to its efficiency. Firstly, it leads to an effective recruitment of NK cells and macrophages which results in strong ADCC and ADCP (picomolar range) [62,63]. Secondly and most importantly, the Fc fragment contributes to the co-stimulation of T-cells through direct contact with macrophages or DC (B7/CD28, CD40/CD40L, LFA3/CD2) and cytokine release (IL-2, IL-6, IL-12).

In a multicenter prospective randomized phase 2/3 study, 258 cancer patients with recurrent symptomatic malignant ascites secondary to epithelial cancers were randomized to compare the intraperitoneal infusion of catumaxomab plus paracentesis with paracentesis alone in order to define its efficacy and safety [85]. This study showed that catumaxomab treatment significantly prolonged puncture free survival (drainage of fluid from the abdomen) (median 46 days) and decreased ascites symptoms in comparison to only paracentesis (median 11 days). Consequently, the European commission approved catumaxomab in April 2009.

Additionally, benefits have been shown in gastric cancer with a diminution of time to next paracentesis and a prolonging of overall survival (77 *versus* 44 days). Catumaxomab has also been shown to eliminate cancer stem cells present in the peritoneal fluid of ascites of patients suffering from advanced ovarian, gastric or pancreatic cancers [86]. Moreover, catumaxomab has demonstrated an acceptable safety profile [85,87] with adverse effects associated with its immunological mode of action remaining manageable and reversible. Interestingly, the development of HAMAs reported in the literature [88,89], in response to the administration of murine/rat Triomab, did not affect the efficiency of catumaxomab in the clinic [87]. On the contrary, the moderate development of antibodies against non-humanized mouse/rat antibody catumaxomab was associated with longer median survival [90].

Catumaxomab is currently evaluated in clinical trials for ovarian cancer, non-small cell lung cancer and malignant pleural effusions (Figure 1) [91].

##### Blinatumomab/Blincyto™

Blinatumomab/Blincyto™ (also known as AMG103 or MT103) is an anti-CD19 × CD3 BiTE developed and licensed by Amgen for relapsed or refractory B-cell precursor acute lymphoblastic leukemia (R/R ALL) treatment (Figure 1). CD19 has a prominent place among the potential tumor target as it is expressed on almost all B-cell malignancies but not on plasma cells nor on hematopoietic stem cells. ALL is a malignant disease of the bone marrow affecting B-cell precursors that is classically treated with intensive chemotherapy. Most of treated patients respond to treatment with complete clinical remission. However, up to 50% of these patients will relapse with a chemoresistant disease. In these cases, treatment options are limited and the chance of survival are very low [92,93].

Numerous *in vitro* studies demonstrated that an anti-CD19 × CD3 BiTE triggers a high cytotoxic response specific of CD19^+^ B-cells through the formation of lytic immune synapses with a molecular topography similar to the natural cytolytic synapses induced by TCR/peptide antigen/MHC class I molecules interactions [79]. This response does not require the engagement of co-stimulatory receptors (e.g., CD28) and is independent of the TCR specificity, leading to a high antitumor activity *in vivo* [64,94,95]. These results have shaken up the classically accepted requirement of co-stimulation signals for full cytotoxic T-cells activation. One explanation might be that BiTEs activate only unstimulated effector/memory T-cells [95]. Another explanation could be that BiTEs induce very close cell/cell contacts leading to the efficient formation of lytic synapses. In line with this hypothesis, the epitope distance to the targeted cell surface was described to play a key role on the effectiveness of BiTEs [96].

Pharmacokinetic studies demonstrated that continuous intravenous infusion via a portable mini-pump is required to overcome the short blood half-life of blinatumomab and its high clearance. In a dose-escalation phase I study, major responses were observed at very low dose of blinatumomab, *i.e.*, a serum level of 0.6 ng/mL (at 0.015 mg/m^2^ per day), about 5 orders of magnitude below serum levels reported for the monoclonal antibody rituximab at a dose of 375 mg/m^2^ per week [97]. Very promising results were obtained in a single arm, open-label, multicenter phase 2 study (protocol MT103-211) in which blinatumomab was given continuously by intravenous infusion for 1 to 5 cycles of “4 weeks treatment-2 weeks break” [98]. This study was conducted with 189 adult R/R ALL patients and the primary endpoint was complete remission (CR) or CR with partial hematologic recovery (CRh) within the 2 first cycles. Both the median relapse-free survival (RFS) and the median overall survival were increased (5.9 months and 6.1 months, respectively) and 43% (CR + CRh) of patients exhibit complete remission, confirming the antitumor activity of blinatumomab. Notably, frequent and serious toxicities (cytokine release syndrome and nervous system disorders) associated with blinatumomab treatment were observed but they were manageable and moderated by dose interruption, conferring an acceptable safety profile. Together, these data indicate that blinatumomab works better than other conventional single-agents on all clinical endpoints (CR + CRh, CR and median RFS).

Facing the unfavorable context of R/R ALL and the favorable results of the phase 1 and 2 studies, blinatumomab/Blincyto™ has been granted accelerated approval by the US FDA in December 2014 for the treatment of Philadelphia chromosome-negative R/R ALL, making it the first bsAb approved by the FDA.

Several clinical trials are ongoing to evaluate the efficacy of blinatumomab *versus* standard care chemotherapy or in other indications such as relapsed/refractory Philadelphia positive B precursor ALL (Figure 1) [99].

#### 3.1.2. In the Pipeline

Driven by these successes, several other bispecific formats targeting CD3 have been generated and most of them have already been demonstrated activity in preclinical studies.

Two others Triomabs are currently in clinical investigations (Figure 1): ertumaxomab (Rexomum^®^) is in a phase 2 for the treatment of metastatic breast cancer (NCT01569412) and Lymphomum (FBTA05) is in a phase 1 for BCL therapy (NCT01138579).

Three other BiTEs are also in phase 1 clinical investigations: (i) an anti-CEA × CD3 (AMG 211, MT111, MEDI-565) for gastrointestinal adenocarcinoma treatment (NCT01284231), developed in collaboration with Medimmune; (ii) an anti-PSMA × CD3 (MT112 BAY 2010112, NCT01723475), for prostate cancer developed by Bayer; and (iii) Solitomab, an anti-EpCAM × CD3 (AMG 110, MT110) for colorectal, lung and gastrointestinal cancer (NCT00635596) [100].

In addition, two anti-CD19 × CD3 are currently developed for the treatment of hematologic malignancies. The more advanced is AFM11, a TandAb by Affimed GmbH which is in phase 1 (NCT02106091). Interestingly, a side by side comparison between AFM11 and a BiTE-blinatumomab construct was made by Reusch *et al.* [101]. AFM11 possesses the same target specificities as blinatumomab but it is bivalent for both CD19 and CD3. AFM11 which has a 100-fold higher affinity for CD3, exhibited a more potent anti-tumor activity *in vitro*, *in vivo* and *ex vivo*, using realistic settings in term of effector to target ratio.

The second one is an anti-CD19 × CD3 DART developed by MacroGenics. Preclinical data revealed an enhanced redirected T-cell killing against CD19^+^ cells by this bsAb compared to anti-CD19 × CD3 BiTE and a potent antitumor activity in mice and chimpanzees [95,102,103]. Deriving from this DART, a CD19 × CD3 DART-Fc (MGD011) is currently developed by MacroGenics in collaboration with Jansen Biotech Inc. (Horsham, PA, USA) in which a Fc moiety has been added for extending its pharmacokinetic properties [104]. To our knowledge, there is no bibliographic data available on this format, yet.

An anti-CD19 × TCR DART targeting an invariable region of TCRαβ has also shown high killing properties against CD19^+^ cells *in vitro* and potent antitumor activity in mice [105], demonstrating an alternative to target T-cells. Two other DART molecules targeting T-cells and different TAA are currently evaluated by MacroGenics in collaboration with Servier (Suresnes, France), MGD006 (also encoded as S80880 by Servier), an anti-CD123 × CD3 in phase 1 (NCT02152956) for the treatment of acute myeloid leukemia (AML) and myelodysplatic syndrome and MGD007, an anti-gpA33 × CD3, in phase 1 for primary and metastatic human colorectal cancers (NCT02248805) (Figure 1).

Besides AFM11 described above, two TandAbs are in the pipeline of Affimed: an anti-EGFRvIIIxCD3/CD16A (AFM21) for solid tumor and an anti-CD33 × CD3 for acute myeloid leukemia [106].

RO6958688, also called CEA-TCB for CEA T-cell bispecific format, is a 2:1 CrossMab format bivalent for CEA and monovalent for CD3. Moreover, mutations have been introduced in the Fc fragment to suppress its effector function [71]. It is currently being investigated in a dose-escalation phase 1 clinical study by Roche for the treatment of CEA positive solid tumors (clinical trial NCT02324257).

Of note, bsAbs of the most basic format, *i.e.*, chemical cross-linking of full size monoclonal antibodies, yielded some interesting results in clinical studies. These types of constructs are being used to “arm” patient’s T cells that have been massively expanded and IL-2 activated prior to re-injection. This approach, called armed activated T cells, presents the advantage of avoiding a direct systemic injection of large amounts of bsAb in the patient, thereby significantly minimizing toxicity issues. Several constructs are being clinically evaluated including HER2Bi (OKT3 × trastuzumab) for the treatment of metastatic breast cancer, CD20bi (OKT3 × rituximab) for the treatment of non-Hodgkin’s lymphoma, and an anti-CD3 × GD2 bsAb for the treatment of relapsed/refractory neuroblastoma [107].

Academic research is not outdone with CD3 targeting bispecific antibodies, with several publications exploring new formats or improving existing formats released each year. Without claiming to be exhaustive, one can cite some formats published recently: (i) s-Fab combined a single-domain anti-tumor and a Fab anti-CD3 [108], (ii) (X)-3s designed by the Dock and Lock™ method and made of an anti-CD3 scFv covalently associated to a dimer of anti-tumor Fabs [109], (iii) other IG-like formats targeting CD20 and CD3 such as REGN1979 [110] and CD20-TDB for T cells-dependent bispecific antibodies [111].

### 3.2. Other Ways to Recruit Effector T Cells

#### 3.2.1. γδ T-Lymphocyte, Potential Targets?

Another way to recruit T-cells is to target specific subset of T-cells. Recently, γδ T-cells arouse a great interest in the tumor immunotherapy field. These unconventional T-cells, actors of the innate immunity, represent only a minor proportion of the peripheral CD3^+^ T cells (1%–5%) [112] but constitute a major subset (20%–50%) of T-cells in epithelial tissues. Circulating γδ T-cells mainly express heterodimers of Vγ9 and Vδ2 chains [113] whereas tissue γδ T-cells preferentially express Vδ1 chains associated with different Vγ chains [114]. These two γδ T-cells subsets display different specificities: some Vδ1 γδ T-cells recognize MICA, MICB and ULBPs molecules which are natural ligands of NKG2D expressed on the surface of stressed epithelial cells while Vδ2Vγ9 γδ T-cells recognize phosphoantigens (PAgs) in a TCR-dependent and MHC-non restricted manner [115,116].

In humans, γδ T-cells are endowed with potent anti-tumor functions (high cytotoxicity and interferon γ secretion) but in some context, they have also been described to play a pro-tumor role through the production of IL-17 [117]. Moreover, Vγ9Vδ2 T-cells can behave as efficient antigen-presenting cells for αβ T-cells and induce adaptive immune responses [115].

Several recent pre-clinical studies have demonstrated that Vδ2Vγ9 T-cells-mediated cytotoxicity is potentiated by the presence of monoclonal antibodies such as rituximab [118,119,120], trastuzumab [119,120] or alemtuzumab [119] through CD16-mediated ADCC.

γδ T-cells have been shown to infiltrate tumors in many cancers but the clinical relevance of their presence is debated due to conflicting results [114]. With the growing data on γδ T-cells, it became evident that their potent anti-tumor activity and their particular features (HLA-non restricted tumor cell killing and reduced of graft-versus-host disease [121]) should be exploited for cancer immunotherapy. Up to now, all the research efforts have been focused on Vδ2Vγ9 LTγδ, and mainly aimed at activating γδ T-cells *in vivo* or *ex vivo* for adoptive transfer. Recently, a [(HER2)_2_ × Vγ9] tribody able to recruit human Vγ9 LTγδ to HER2-expressing malignant cells has been generated [121,122]. This bispecific tribody is composed of two HER2-specific scFv fused to an anti-Vγ9 Fab. The authors have shown that [(HER2)_2_ × Vγ9] was more efficient than PAg at triggering the cytotoxic activity of circulating γδ T-cells against pancreatic ductal adenocarcinoma (PDAC) cell lines [122] as evidenced by a higher release of perforin and granzyme B. Moreover, [(HER2)_2_ × Vγ9] provoked less cell death induction in activated γδ T-cells lines than PAg, an interesting property in the perspective of adoptive transfer. A side by side comparison between [(HER2)_2_ × Vγ9] and [HER2 × CD3] demonstrated that both molecules improved the cytotoxic potential of PAg-activated γδ T-cells but only [(HER2)_2_ × Vγ9] was able to enhance the cytotoxicity of freshly isolated γδ T-cells. Altogether, these results led the authors to propose [(HER2)_2_ × Vγ9] tribody as an attractive and potent PAg alternative for γδ T-cells -based cancer immunotherapy (Figure 2).

Although clinical studies are not yet abundant, preliminary data highlight the importance of taking into account the γδ T-cells subset in T-cell-based immunotherapy.

#### 3.2.2. Targeting Inhibitory Receptors?

The physiological role of immune checkpoints, molecules involved in the regulation of immune cells, is to maintain the immune homeostasis to prevent autoimmunity or tissue injury. However, in tumor settings, they can be hijacked by tumor cells to elude the anti-tumor activity of the immune system. The idea of targeting these molecules represents a groundbreaking milestone for cancer immunotherapy. Inhibition of immune checkpoints implicated in immune cells exhaustion inside the tumors such as cytotoxic T-lymphocyte antigen 4 (CTLA-4) or PD-1/PD-L1 pathway has led to durable clinical responses in many cancers and in some cases to long-term remission. The main downsides reside in the response heterogeneity among patients and a manageable level of autoimmune toxicity. Currently, three immunomodulatory antibodies targeting CTLA-4 (ipilimumab (Yervoy^®^; Bristol-Myers Squibb)) and PD-1 (nivolumab (Opdivo^®^; Bristol-Myers Squibb), and pembrolizumab (Keytruda^®^; Merck Sharp & Dohme Corp)) have gained accelerated approval by the U.S FDA for metastatic melanoma and non-small cell lung cancer (Keytruda^®^) but no doubts that they will be soon accepted for other types of cancer (for a detailed review see [123]). An army of studies are underway on the development of inhibitors against other known immune modulating molecules such as LAG3, TIM3 or CD137, on combination approaches for expanding efficacy and on the discovery of tumor response rate markers. The impressive clinical successes recorded with these approaches pave the way for strategies based on bispecific antibodies for tackling refractory tumors (Figure 2). In 2014, a partnership between AnaptysBio (San Diego, CA, USA), a therapeutic antibody company, and TESARO Inc. (Waltham, MA, USA), a company dedicated to oncology therapeutics, was initiated for developing two bispecific antagonist antibodies targeting PD-1/TIM-3 and PD-1/LAG-3 using SHM-XEL (somatic hypermutation/mammalian cell system platform) [124].

In 2015, in the course of the 37th San Antonio Breast Cancer Symposium, Sorrento Therapeutics Inc. (San Diego, CA, USA), another clinical-stage biopharmaceutical company, communicated on a chemical IgG-like bispecific antibody (CBA-0710) directed against PD-L1 and c-Met. This bsAb demonstrated *in vitro* efficient immunomodulation functions in a breast cancer model [125,126]*.*

Despite scarce data for now, these new immune checkpoint bispecific approaches are likely in pipeline of several pharmaceutical companies, extending the field of applications of bispecific antibodies. There is no doubt that the near future will see a blossoming of these bispecific formats.

## 4. Innate Immunity in bsAb-Mediated Immunotherapy

Besides the concept of T-cell targeting by bispecific antibodies, other attractive approaches that aim at leveraging the innate immune system are slowly gaining ground with pharmaceutical companies.

### 4.1. NK Cells

NK cells are classically divided into two subsets: CD3^−^CD56^bright^CD16^low^ and CD3^−^CD56^dim^CD16^bright^. The vast majority of NK cells circulating in the blood, display a CD3^−^CD56^dim^CD16^bright^ phenotype and a potent cytotoxic activity. A minor subset belonging to the CD3^−^CD56^bright^CD16^low^ phenotype and residing in lymph nodes and tonsils displays low cytotoxic activity but produces a variety of cytokines and chemokines. Circulating NK are mostly in a non activated state but become able to penetrate tissues containing infected or transformed cells upon cytokines-mediated activation.

NK cells exert their cytotoxic effect by several mechanisms: (i) direct cytotoxic activity through their capacity to discriminate between self and “non self or stress-induced self” using their wide array of activating receptors (NKG2D and natural cytotoxic receptors (NKp30, NKp44, NKp46)) and inhibitory receptors (KIR, NKG2A); (ii) antibody dependent cytotoxic activity (ADCC) through FcγRIIIA receptor (CD16A) engagement by antibody-coated target cells; (iii) secretion of chemokines and cytokines such as interferon γ that participate to shape innate and adaptive immune responses [127].

The clinical importance of NK cells in oncology has been evidenced by several studies that report an association between the cytotoxic status of peripheral NK cells and an increased cancer risk [128,129] as well as between the presence of tumor infiltrating NK and a better prognosis [130,131,132]. Moreover, recent studies have highlighted the crucial role of cytotoxic NK cells in the killing of cancer cells displaying a cancer stem cell phenotype *in vitro* and *ex vivo.* However, this aptitude is often lost in the tumor environment in favor of an acquired ability to produce cytokines, a phenomenon called “split anergy” [133].

NK cell-based therapeutic approaches have been intensively explored through (i) adoptive transfer of NK cells [134,135,136] derived from different sources (peripheral or cord blood, hematopoietic or embryonic stem cells) [137]; (ii) genetically modified NK cells expressing chimeric antigen receptor (CAR) [137]; or (iii) antibody based immunotherapy.

Several lines of evidence support ADCC as a pivotal mechanism underlying the anti-tumor efficacy of several therapeutic mAbs such as rituximab, trastuzumab or even checkpoint inhibitor ipilimumab which exerts its efficacy through both immune check point inhibition and ADCC [132,138]. As a result, tremendous efforts have been made to enhance the effector function of therapeutic mAb using different approaches such as fucose removal and Fc mutations [45,46].

Another attractive approach is the design of bispecific antibodies that aim at recruiting and activating NK cells to tumor site via the potent CD16A receptor (Figure 2).

#### 4.1.1. From Bench to Clinic: Only One Bispecific Antibody

Up to now, only one bispecific antibody, with ADCC as unique mode of action, is currently in clinical trial (NCT02321592) (Figure 1). Indeed, AFM13, an anti-CD30 × CD16A TandAb developed by Affimed, represents the first-in-class tetravalent bsAb for the treatment of Hodgkin’s disease [139]. Preclinical studies showed a very efficient anti-tumor activity of AFM13 with no off-target NK cell activation despite the bivalent binding to CD16A. A phase1 dose-escalation study (NCT01221571) involving 28 patients has demonstrated that AFM13 has an acceptable safety profile [139] and a phase 2 clinical investigation (NCT02321592) is ongoing to evaluate the efficiency of AFM13 for the treatment of patients with relapsed or refractory Hodgkin lymphoma. On the same model, an anti-EGFRvIII × anti-CD16A TandAb, recruiting NK cells is currently in the pipeline of Affimed.

#### 4.1.2. In the Pipeline

Diverse bispecific formats designed to deplete target cells through CD16-mediated ADCC are reported in the literature. The following non-exhaustive list presents some promising constructs.

The best characterized formats are bispecific/trispecific Killer Engagers (BiKE and TriKE). They are multispecific antibodies made of 1 or 2 scFvs targeting tumor associated antigen and one scFv targeting CD16A, connected via a small fragment of human muscle aldolase. Gleason *et al.* [140] demonstrated the ability of two such constructs, CD16 × 19 BiKE and CD16 × 19 × 22triKE, to recruit and enhance NK cell effector function through direct CD16 signaling. More recently, a CD16 × 33 BiKE has been reported by the same group for the treatment of myelodysplatic syndromes (MDS) [141]. They have shown that, despite a lower expression of CD16 in MDS NK, CD16 × CD33 BiKE successfully mediated the killing of CD33^+^ cells. Constructed on the same model, although not called BiKE, a bispecific antibody called EpCAM16, targeting EpCAM and CD16 turned out to be capable of boosting NK-mediated killing of several EpCAM^+^ tumors [142].

Schubert *et al.* [143] developed several single chain triplebodies (sctb) corresponding to three scFvs in tandem. In these constructs, the two distal scFvs target TAA while the central scFv targets effector immune cells. This group has designed a disulfide stabilized 33-ds16-ds19 triplebody (ds for disulfide bond between VH-VL of the scFv) targeting CD33 and CD19, myeloid and lymphoid markers respectively, co-expressed on mixed lineage leukemia cells [143]. This format was shown to be more reliable than the control formats (33-ds16 and ds19-ds16) and to efficiently mediate tumor cells lysis in this pathological context [143].

Based on a CL-CH1 heterodimerization scaffold [144] similar to that described by Schoonjans *et al.* [145], Lu *et al.* [146] have developed a bispecific trivalent antibody made of two scFv targeting HER2 and one Fab fragment targeting CD16A. This format demonstrated an anti-tumor superiority compared to an HER2 scFv-Fc fusion both *in vitro* and in a tumor xenografted mice model [146].

Our team has designed Fab-like bispecific antibodies (bsFabs) [147,148] using llama single-domain antibodies (sdAbs or VHH) and the natural affinity of human CH1 and Cκ IgG domains as heterodimerization motif. Llama single-domain antibodies, derived from heavy-chain antibodies naturally devoid of light chains [149], are endowed with unique structural and functional properties. They are small (13 kDa), stable and easily produced. Their high identity to the human type 3 V_H_ domains [150,151] should elicit low immunogenicity in human, and facilitates humanization if needed [152]. Moreover, through their small size and single domain nature, sdAbs can recognize epitopes usually not accessible to conventional antibodies. Thus, sdAbs represent ideal molecular building units for the construction of stable bsAb devoid of hydrophilic linkers [153]. We have exploited the bsFab format to produce a HER2 × CD16 bsFab (HER2bsFab) targeting binding sites on HER2 and CD16 different from those targeted by trastuzumab and Fc fragment. HER2bsFab has a moderate affinity for HER2 (60 nM) and a unique and high affinity specific for CD16 (36-fold higher than trastuzumab). A side by side comparison between HER2bsFab and trastuzumab revealed that HER2bsFab triggered a stronger NK-mediated ADCC than trastuzumab against HER2^low^ and trastuzumab-refractive cell lines. Interestingly, *in vivo*, HER2bsFab was able to inhibit HER2^low^ tumor growth with a net superiority over trastuzumab. Moreover, the CD16-engagement by HER2bsFab was independent of CD16 V/F158 polymorphism and induced a stronger NK cells activation than trastuzumab in response to target cell recognition. Thus, this molecule has the potential to enlarge the number of patients eligible for breast cancer immunotherapy and to consider therapeutic combination strategies.

### 4.2. Macrophages

Macrophages are innate immune cells involved in tissue homeostasis and host defense. They derive from circulating monocytes that extravasate from blood vessels and differentiate into macrophages in the peripheral tissue under the action of macrophage colony-stimulating factor (M-CSF). It has been shown that macrophage recruitment by the tumor starts at the very beginning and persists all along the development of the tumor [154]. In the tumor microenvironment, tumor cell-derived factors (e.g., TGF-β1, IL-10, IL-6) induce the polarization of macrophages into tumor-associated macrophages (TAMs) with pro- or anti-tumor phenotypes. The preponderant phenotype observed in tumor stroma is a tumor promoting phenotype characterized by the expression of molecules promoting angiogenesis (e.g., VEGF-C, IL-8), tumor growth (TNF-α) and invasiveness (MMP-7, MMP-9) [155,156]. Additionally, TAMs are endowed with immunosuppressive properties through the release of diverse molecules leading to the attraction of T_reg_ cells and stimulation of PD-L1 expression on their surface inducing T cells dysfunction [157,158]. Clinical data have shown that TAMs constitute a major part of immune infiltrates and are generally associated with tumor growth [159] and poor clinical outcome in various cancers [160,161,162,163].

Although marginalized due to the complex TAMs/tumor relationships, macrophages may also exert an anti-tumor activity through direct tumor cell killing induced by “eat me” signals such as exposure of phosphatidyl serines, changes in glycosylation of surface molecules or in surface charge. They also use antibody-dependent cellular phagocytosis (ADCP) triggered by Fc/FcγR interactions, a process which has been shown to play a critical role in the efficacy of therapeutic antibodies [164,165,166]. This may be explained by the large panel of Fcγ receptors present on the surface of macrophages in contrast to NK cells which express only FcγRIIIA.

Consequently, many strategies have been developed to improve macrophage engagement including antibody engineering to increase their affinity for Fcγ receptors [167] and the design of bispecific antibodies to cross-link macrophages and tumor cells (Figure 2). Of note, all the above mentioned bispecific antibodies targeting CD16A are likely able to trigger macrophage-mediated ADCP but related data are scarce.

As macrophages express all classes of Fcγ receptors, early works investigated the potential of FcγRI-dependent signaling. The anti-HER2 × FcγRI bispecific antibody MDX-210 was a chemically cross-linked bispecific Fab format generated for the treatment of advanced breast or ovarian cancer that over-expressed HER2 [168]. *In vitro* studies showed that MDX-210 was capable of inducing phagocytosis [169], but clinical investigations demonstrated that MDX-210 and its partially humanized version (MDX-H210) achieved only modest clinical benefits [168,170]. Equally disappointing results have been obtained with an anti-EGFRxFcγRI bispecific antibody [171].

A conceptually different strategy related to the checkpoint inhibitor concept holds much promise. It is based on the modulation of macrophage-mediated phagocytosis by the CD47/SIRPα myeloid-specific inhibitory checkpoint. Although expressed on almost all human cells, CD47 is overexpressed in many cancers and has been shown to inhibit macrophage phagocytosis through its interaction with the signal regulatory protein α (SIRPα) present on macrophages. Preliminary clinical data demonstrated that CD47-blocking therapies strongly enhanced the potential of macrophages to destroy tumors [172]. Recently, an anti-CD20 × CD47 bispecific antibody was designed for the treatment of non-Hodgkin lymphoma (NHL) [173], using the dual-variable-domain immunoglobulin (DVD-IgG) format [174]. *In vitro* studies have shown that this anti-CD20 × CD47 bsAb, through tumor targeting and CD47 blockade, guided the macrophage attack and induced an efficient phagocytosis of malignant cells in a FcγR-dependent manner, which translated *in vivo* by a reduced lymphoma burden and an increased mice survival [173].

## 5. Conclusions

The emergence of next-generation antibody therapeutics such as bispecific antibodies, antibody-like proteins or Fc-engineered antibodies and the loss of exclusivity of several lead antibodies (trastuzumab, rituximab for instance) in the next few years will likely re-shape the antibody market landscape. Although the “bispecific” market is still emerging, it is moving fast and is already composed of a rich and diversified pipeline with promising therapeutic potential to take up immunotherapy challenges.

Indeed, since the first generation of bispecific antibodies, advances of antibody engineering technologies and deeper understanding of the immuno-oncology field have enabled bispecific antibodies to enter the clinic as a class of antibody-based therapeutics in its own right for cancer treatment, as evidenced by the recent approvals of Removab^®^ and Blincyto™. Beside the evident attractivity of T-cell triggering, the encouraging clinical results of bsAb retargeting NK cells or other effector cells appreciably enlarges the spectrum of strategies to recruit, boost, modulate or awake the immune system in tumor settings.

Supported by the intrinsic flexibility of bsAb for creating a diversity of dual targeting strategies, the field of bispecific antibodies brings innovative solutions that are poised to deliver exciting results in a near future.
